# Plasma biomarkers in neuropsychiatric syndromes: A narrative review

**DOI:** 10.1177/13872877251392185

**Published:** 2025-11-24

**Authors:** Zahinoor Ismail, Dylan X Guan, Ganesh M Babulal, James R Bateman, Marc Cantillon, Byron Creese, Fabrizia D'Antonio, Corinne E Fischer, Jennifer R Gatchel, Maryam Ghahremani, José Guerrero-Cantera, Krista L Lanctôt, Michelle M Mielke, Moyra E Mortby, Oriel Feldman, Ana C Pereira, Arturo X Pereiro, George Perry, William Z Potter, Miriam Rabl, Ramit Ravona-Springer, Allyson C Rosen, Hillary J Rouse, Krushnaa Sankhe, Suzanne E Schindler, Chinedu T Udeh-Momoh, Rawan Tarawneh, Fabricio Ferreira de Oliveira, for the Alzheimer’s Association International Society to Advance Alzheimer’s Research and Treatment (ISTAART), Biofluid Based Biomarkers in Neuropsychiatric Syndromes work group

**Affiliations:** 1 Hotchkiss Brain Institute, University of Calgary, Calgary, Alberta, Canada; 2 Department of Psychiatry, University of Calgary, Calgary, Alberta, Canada; 3 Department of Clinical Neurosciences, University of Calgary, Calgary, Alberta, Canada; 4 Department of Community Health Sciences, University of Calgary, Calgary, Alberta, Canada; 5 O’Brien Institute for Public Health, University of Calgary, Calgary, Alberta, Canada; 6 Department of Pathology and Laboratory Medicine, University of Calgary, Calgary, Alberta, Canada; 7 Clinical and Biomedical Sciences, Faculty of Health and Life Sciences, University of Exeter, Exeter, UK; 8 Department of Neurology, Washington University School of Medicine, St Louis, MO, USA; 9 Institute of Public Health, Washington University School of Medicine, St Louis, MO, USA; 10 Department of Psychology, University of Johannesburg, Johannesburg, South Africa; 11 Department of Neurology, Wake Forest University School of Medicine, Winston-Salem, NC, USA; 12 Department of Psychiatry, Robert Wood Johnson Medical School, New Brunswick, NJ, USA; 13 Department of Psychology, Brunel University of London, London, UK; 14 Department of Human Neuroscience, Sapienza University of Rome, Rome, Italy; 15 Cognitive and Motor Rehabilitation and Neuroimaging Unit, IRCCS Fondazione Santa Lucia, Rome, Italy; 16 Keenan Research Centre for Biomedical Science, Li Ka Shing Knowledge Institute, St Michael's Hospital, Toronto, Ontario, Canada; 17 Massachusetts General Hospital, Harvard Medical School, Boston, MA, USA; 18 McLean Hospital, Harvard Medical School, Belmont, MA, USA; 19 Instituto Mexicano del Seguro Social, Mexico City, Mexico; 20 Hurvitz Brain Sciences Program, Sunnybrook Research Institute, Toronto, Ontario, Canada; 21 Department of Psychiatry, University of Toronto, Toronto, Ontario, Canada; 22 Department of Epidemiology and Prevention, Wake Forest University School of Medicine, Winston-Salem, NC, USA; 23 School of Psychology, UNSW Sydney, Sydney, New South Wales, Australia; 24 Neuroscience Research Australia, Sydney, New South Wales, Australia; 25 UNSW Ageing Futures Institute, UNSW Sydney, Sydney, New South Wales, Australia; 26 Department of Neurology, Icahn School of Medicine, Mount Sinai, NY, USA; 27 Cognitive Neuroscience Research Group, Health Research Institute of Santiago de Compostela, Santiago de Compostela, Spain; 28 Instituto de Psicoloxía (IPsiUS), Universidade de Santiago de Compostela, Santiago de Compostela, Spain; 29 Department of Neuroscience, Developmental and Regenerative Biology, University of Texas at San Antonio, San Antonio, TX, USA; 30 Independent Consultant, Philadelphia, PA, USA; 31 Department of Psychiatry and Psychotherapy, Psychiatric University Hospital Zurich, University of Zurich, Zurich, Switzerland; 32 The Joseph Sagol Neuroscience Center, Sheba Medical Center, Tel HaShomer, Israel; 33 Memory Clinic, Sheba Medical Center, Ramat Gan, Israel; 34 Faculty of Medical and Health Sciences, Tel Aviv University, Tel Aviv, Israel; 35 VA Medical Center-Palo Alto, Palo Alto, CA, USA; 36 Stanford University, School of Medicine, Stanford, CA, USA; 37 School of Aging Studies, University of South Florida, Tampa, FL, USA; 38 Department of Psychiatry and Pharmacology/Toxicology, University of Toronto, Toronto, Ontario, Canada; 39 Department of Neurology, Washington University School of Medicine, St Louis, MO, USA; 40 School of Public Health Sciences, Wake Forest University School of Medicine, Winston-Salem, NC, USA; 41 Brain and Mind Institute, Aga Khan University, Nairobi, Kenya; 42 Division of Clinical Geriatrics, Center for Alzheimer Research, Karolinska Institutet, Stockholm, Sweden; 43 Sheffield Institute for Translational Neuroscience (SITraN), University of Sheffield, Sheffield, UK; 44 Department of Neurology, Saint Louis University, St Louis, MO, USA; 45 Escola Paulista de Medicina, Universidade Federal de São Paulo, São Paulo, Brazil

**Keywords:** Alzheimer disease, behavioral symptoms, cognitive disorders, frontotemporal dementia, Lewy body disease, mild behavioral impairment, mild cognitive impairment, neurodegenerative diseases, neuropsychiatry, vascular cognitive impairment

## Abstract

Neuropsychiatric symptoms (NPS) are common features of neurodegenerative disease (NDD) but are relatively understudied compared to cognition, especially regarding biomarkers. Further, emerging evidence describes the utility of systematic assessment of NPS across the cognitive continuum, even in advance of dementia. In this narrative review, we discuss the role of plasma biomarkers in relation to NPS across the cognitive continuum of unimpaired, subjective cognitive decline, mild cognitive impairment, and dementia. While Alzheimer's disease is the primary focus, vascular, Lewy body, and frontotemporal dementia etiologies are also discussed. Literature searches included NPS and dementia-related search terms with additional literature identified based on the author group's subject area expertise. We found that plasma biomarkers are a burgeoning field, and scalability and accessibility make them well-suited for the study of NPS across the disease continuum. In early-stage NDD, diagnostic biomarkers are best suited for discriminating NDD-related NPS from non-NDD psychiatric syndromes and/or NPS due to other causes. In those with dementia, monitoring and prognostic biomarkers may enable the assessment of treatment response or help predict the risk of worsening symptoms. We conclude that plasma amyloid-β and tau show great promise in assessing NPS, especially during early-stage disease, but inflammatory and genetic biomarkers may also play a role across the disease course. Systematic research is required, keeping in mind the ethical considerations of knowing biomarker status in early-stage disease.

## Introduction

Disease-specific biomarkers may assist in patient diagnosis, improving accuracy over clinical assessment alone, and accelerating therapeutic delivery.^1^ Mechanistic biomarkers can inform understanding of disease, and are important for diagnostics and therapeutics.^2^ However, even autopsy-based classification criteria are based on the quantity of pathology for a given age in the presence of dementia and only at a specific point in time^3^; thus, the traditional sense of “gold standard” may not apply.^4^ For Alzheimer's disease (AD), the most common cause of neurodegenerative disease (NDD), some frameworks define AD on the basis of pathology alone, whether or not clinical symptoms are present. In contrast, other frameworks define AD based on both pathology and clinical symptoms.^5,6^ Notably, both AD pathology and cognitive impairment become very common after the age of 85 years.^7,8^ Similarly, efforts are underway to incorporate biomarkers to redefine Lewy body diseases, especially in early stages,^9^ and in vascular cognitive impairment.^10,11^ Frontotemporal dementias are also under investigation, but disease heterogeneity provides challenges in identifying specific but broadly applicable biomarkers.^12^

In AD, complicating the diagnostic issue is dynamic change of biofluid amyloid-β (Aβ) biomarkers throughout the disease course. Amyloid pathology accumulates in the brain for 10–15 years while individuals remain clinically unimpaired, but high amyloid levels are associated with a higher risk of developing cognitive impairment.^13,14^ Cerebrospinal fluid (CSF) Aβ_42_ declines even before amyloid plaques are apparent on brain imaging,^15^ and a low Aβ_42/40_ ratio may reflect soluble protofibrils more closely than amyloid plaques.^16^ Fluid biomarkers, including tau, phosphorylated at positions 181, 217, and 205 (p-tau_181_, p-tau_217_, and p-tau_205_, respectively), change later than Aβ_42/40_ in the course of the disease and are more strongly associated with cognitive symptoms.^17–20^ A CSF peptide, including the microtubule-binding region of tau (MTBR-tau_243_), changes even later in the course of AD and has the highest associations with tau and cognitive symptoms demonstrated thus far by a fluid biomarker.^21^ But how do neuropsychiatric symptoms (NPS) or behavior fit into this time course?

Incorporating NPS into this biological understanding of the initiation and early stages of AD may increase complexity but may also reveal the physiological basis of behavioral change in AD.^22^ Behavioral variants have not been systematically evaluated in association with mechanistic biomarkers. Biomarkers can unlock critical insights in pathogenesis in addition to diagnosis. The complexity of change in biomarkers with dementia onset supports a pivotal and integrated biological role for Aβ, neurodegeneration, oxidative damage, and mitochondria in the transition to AD.^23,24^

The purpose of assessing biomarkers in patients with dementia is to focus on marking treatment response or assessing risk of worsening symptoms so that intervention strategies can be developed, i.e., monitoring, prognostic, and predictive biomarkers.^25^ Here, it is unlikely that AD diagnostic biomarkers will be useful. In particular, Aβ accumulation can plateau as cognitive impairment worsens, meaning that sensitivity in detecting NPS prevalent in late-stage neurodegeneration may be lost, and p-tau_205_ or MTBR-tau_243_ may be better markers at later stages.^2,18,21^ Biomarkers of dementia progression may provide a way to assess the risk for emergent NPS, since most NPS seem to correlate with a more severe disease course. However, whether these NPS reflect direct mechanisms (i.e., treatment targets) remains at equipoise. Thus, earlier in the disease course (i.e., preclinical and prodromal disease), susceptibility/risk and diagnostic biomarkers may be more appropriate to explore links with NPS to help distinguish between NDD-related NPS and NPS due to other etiologies.^25^ However, examining the relationship between NPS and underlying neuropathology is challenged by variability in the profile and measurement of specific types of NPS^26,27^ and biomarkers, including changes over the course of the disease,^28^ and the age of onset,^29^ type,^30^ severity,^31^ and regional distribution^32^ of the underlying neuropathology.

In this narrative review, we explore associations between NPS and NDD biomarkers across the cognitive spectrum, from cognitively unimpaired (CU) to subjective cognitive decline (SCD), mild cognitive impairment (MCI), and dementia. The primary focus is AD, for which there are the most data, attendant with the high prevalence of AD relative to other dementias.

## Basics of plasma biomarkers

The extent to which any specific analyte measured in blood correlates with its concentration in CSF or brain tissue is highly variable. It depends on multiple processes, including the structure and function of the blood-brain barrier and the rates of synthesis and clearance of analytes in the periphery and the brain.^20,33^ Although many processes involved in steady-state equilibria across tissue compartments have been elucidated, much less is known regarding the extent to which a substance formed in the brain reaches the blood in its original form, particularly given the potential impact of anthropometric, demographic, and genetic features on biofluid levels of any substance.^34^ Preclinical studies of blood biomarkers can be informative, but they are not consistent across species. Thus, how well blood biomarker concentrations reflect brain concentrations, and the impact of peripheral production and clearance rates, is not fully understood. The accepted technique for determining the amounts and rates of brain contribution to the blood in humans is to label the central pool of an analyte by administering a heavy isotope-labeled precursor of the analyte of interest.^35^

Initial studies of Aβ peptides in CSF and blood have demonstrated significant between- and within-immunoassay variation over time. This assay “noise” was greater in blood than in the CSF matrix, obscuring the relationship between plasma Aβ and brain amyloid plaques as detected by higher precision assays.^36^

A major challenge in measuring blood-based AD biomarkers is that the concentrations of Aβ and tau are substantially lower in the blood than in CSF. For example, concentrations of plasma Aβ_42_^37^ and tau^38^ are approximately 30 and 2–5 times lower, respectively, than in CSF. Another challenge is that plasma and serum contain higher total protein concentrations, which can interfere with the measurement of AD blood biomarkers. As a result, the development of blood biomarkers was slowed due to the high sensitivity required, the limited correlation between blood and CSF biomarkers, and the high variability encountered when using conventional enzyme-linked immunosorbent assays or other immunoassays.^39,40^

Technological advances in the last decade have contributed to the development of more accurate assay platforms capable of sensitively measuring Aβ peptides, p-tau proteins, and neurodegeneration biomarkers (neurofilament light chain [NfL] and glial fibrillary acidic protein [GFAP]) in the blood. These platforms include, among others, the Single Molecule Array (Simoa) immunoassay, immunoprecipitation mass spectrometry (IP-MS), Meso Scale Discovery platform, Elecsys immunoassay, and immunomagnetic reduction. An overview of available assays and platforms is available elsewhere.^20^ A head-to-head comparison of eight assays assessing the performance of plasma Aβ_42/40_ for detecting abnormal brain amyloid, as measured by positron emission tomography (PET) and CSF, suggested that IP-MS assays performed better than many of the immunoassays in both cognitively impaired and unimpaired older adults.^41,42^ More recent studies have found that some immunoassays have performance similar to IP-MS assays in some cohorts, supporting that immunoassays may be adequate for certain applications.^43,44^

Head-to-head comparisons of plasma p-tau biomarkers have also suggested that IP-MS assays perform slightly better than immunoassays for predicting Aβ pathology and that p-tau_217_ predicts brain amyloid pathology more strongly than p-tau_181_ or p-tau_231_.^44–48^ However, compared to immunoassays, IP-MS may require more specialized equipment and larger sample volumes, making implementation at the population level more challenging. A recent advancement is the development of Nucleic Acid Linked Immuno-Sandwich Assay (NULISA), which is a multiplex assay allowing testing of 120 NDD-related proteins to attomolar-level sensitivity using small sample volumes.^49–51^ NULISA has demonstrated discriminative accuracy for abnormal amyloid-PET (area under the receiver operator curve (AUC = 0.918)) and tau-PET (AUC = 0.939),^52^ comparable to Simoa,^53^ and potentially easier to implement at the population level than IP-MS. Further research on scalability is required.

### Comorbid conditions affecting the interpretation of AD blood-based biomarkers

AD blood biomarker levels are more susceptible to change due to peripheral factors than their PET and CSF counterparts. Due to physiological mechanisms, these peripheral factors can alter the interpretation of blood-based levels and potentially result in false positive or false negative diagnoses.^54^ Here, we highlight three factors that warrant consideration: chronic kidney disease (CKD), obesity, and cardiac conditions.

Multiple studies have shown that a diagnosis of CKD, low estimated glomerular filtration rate, and high creatinine are associated with higher blood levels of all Aβ peptides, p-tau, NfL, and GFAP.^55–58^ Reduced renal clearance of these proteins has been proposed as the underlying mechanism. Thus, CKD could lead to a false positive diagnosis if a blood p-tau marker is used and a false negative diagnosis if blood Aβ is used. One potential strategy to mitigate the effects of CKD on blood biomarkers is to utilize ratio-based measures. For example, CKD has not been associated with as pronounced a change in blood Aβ_42/40_ or p-tau_217_/tau ratios.^56,57,59,60^

Obesity has been associated with lower levels of the aforementioned AD blood biomarkers,^55–57^^,61^ but not with their CSF counterparts.^61,62^ One explanation is that individuals with obesity have larger blood volumes, resulting in lower concentrations of blood AD biomarkers. Similar to CKD, ratio-based measures may mitigate these effects.^46,56,57,60^

Evidence also suggests associations between blood Aβ levels and cardiac conditions. Aβ can accumulate in the hearts of patients with AD, contributing to AD-related cardiac amyloidosis.^63,64^ Further, plasma Aβ_40_ accumulation in vascular walls and heart tissue has been associated with cardiac dysfunction, coronary heart disease, heart failure, and cardiovascular disease mortality.^65^ Higher plasma Aβ_42_ levels have been associated with a higher risk of heart failure.^66^ Entresto^®^ (combination of sacubitril and valsartan), a drug used for heart failure, has been shown to reduce plasma Aβ_42/40_ but not CSF Aβ_42/40_.^67^ As a result, it remains unclear how best to utilize plasma Aβ measures for screening or diagnosis of AD among individuals with heart conditions. Aβ_40_ can also accumulate in cortical and leptomeningeal vessel walls, as is the case in cerebral amyloid angiopathy (CAA), a progressive cerebral small vessel disease that contributes to morbidity and mortality from hemorrhagic stroke and vascular dementia.^68^

### Standardization and pre-analytic variables

Several sources of variation and pre-analytical factors should be considered when interpreting AD blood-based biomarkers. These factors range from circadian rhythms, dietary influences, and other person-specific variables to the collection and processing of the blood samples.^69^ Pre-analytical factors that may affect levels of blood Aβ, p-tau, total tau, NfL, or GFAP include the collection tube type (e.g., anticoagulant used), time to centrifugation and storage, and storage temperature (Table 1).^70^ Other sources of variability include protein instability following multiple freeze-thaw cycles and the distinct sensitivities of antibodies used in different assays.^69,^^71–74^ Aβ_42_ is sensitive to pre-analytical factors, but even more so is total tau,^70,73^ for which details such as time to centrifugation and centrifugation temperature are essential variables.^70^ Certain factors may mitigate some of this variability, including fully automated technologies, ultrasensitive approaches, multiplexing, and rigorous standards such as those required in Clinical Laboratory Improvement Amendments (CLIA)-certified labs.^69,75^ Wider adoption of standardized pre-analytic protocols across global centers is recommended to ensure consistency, improve the reproducibility of tests, and minimize variability of results.^70^

**Table 1.** Effects of preanalytical factors on plasma AD biomarkers.

Tube Matrix for plasma (anticoagulant): EDTA, heparin, fluoride, or citrate, and Serum	Lithium-heparin versus EDTA	Higher plasma Aβ_40_ and Aβ_42_, lower total tau (Roche-Elecsys)^73^ Higher plasma Aβ_40_ and Aβ_42_ levels (various assays)*^70^ Higher plasma Aβ_42_, Aβ_42/40_ ratio, total tau, p-tau_181_, GFAP, and NfL; no significant differences in Aβ_40_^71^
	Na-citrate versus EDTA	Lower total tau, p-tau_181_, Aβ, NfL, GFAP^70^ Lower plasma Aβ_40_, Aβ_42_, and total tau, Higher Aβ_42/40_ ratio (Roche-Elecsys)^73^ Lower plasma Aβ_40_ and Aβ_42_ levels using various assays*^70^ Lower plasma NfL, GFAP, and higher Aβ_42_ and Aβ_42/40_ ratio; no significant differences in Aβ_40_, tau, or p-tau_181_^71^
	Heparin, citrate, fluoride, or serum versus EDTA	Lower plasma Aβ levels (INNO-BIA)^72^
	Aprotinin addition	No effect on plasma Aβ levels (INNO-BIA)^72^
	Serum	Variable (no effect, higher or lower) compared to plasma EDTA^70^
Tube parameters	Material: PP, PET	No effect on plasma Aβ (INNO-BIA), or plasma Aβ_42_ or Aβ_40_ by various assays*
	Sample volume (mL): 2.7, 4.9, 8.5, 9, 10	No effect^73^
	EDTA cation: K2, K3	No effect^73^ No effect on plasma Aβ (INNO-BIA)^72^
	Gel separator: +/-	No effect^73^
	Tube filling: ½ or full	No effect on any markers^73^ Decrease of total tau in one study
Aliquot volume	250, 500, 1000 µl	No effect on plasma Aβ_42_ and Aβ_40_ by various assays*^70^
Collection	Needle size, location of blood draw, tube collection order	No effect^70^
Centrifugation temperature and force	Centrifugation temperature Force	Plasma Aβ_42_ slightly higher when centrifuged at 20°C compared to 2–8°C (INNO-BIA)^72^ Plasma Aβ_40_ levels slightly lower when centrifuged at 20°C compared to 2–8°C (INNO-BIA)^72^ No effect on plasma Aβ_42_ and Aβ_40_ (various assays)*^70^ Lower total tau levels with centrifugation at 4°C^73^Centrifugation at ≥2000 g favored for optimal recovery of Aβ_40_ (INNO-BIA)^72^
Whole blood stability in EDTA tube	Time to centrifugation (h): 0.5, 1, 2, 6, 24 Time to centrifugation (h): 1, 4, 24, 72 Time to storage	Plasma Aβ_40_ and Aβ_42_ stable up to 1 h; plasma Aβ_42/40_ ratio stable up to 2 h; total tau stable up to 6 h (Roche-Elecsys)^73^
		Plasma Aβ_42_ and Aβ_40_ stable up to 4 h at 20°C and up to 24 h at 2–8°C (INNO-BIA)^72^ Whole blood Aβ_42_ and Aβ_40_ stable up to 24 h at 4°C but not at RT* and decrease afterwards; effects not mitigated by Aβ_42/40_ ratio for most assays*^70^ Plasma GFAP, NfL, and p-tau_181_ levels stable if tubes held >24 h at RT or 4°C^70^ Lower whole blood total tau if held at RT for 3–24 h, and higher if held at 4°C for 3–24 h before centrifugation^70^
		No effect of delay in fresh plasma storage up to 6 h on Aβ_42_, Aβ_40_, or total tau levels 4°C (Roche-Elecsys)^73^ Lower plasma total tau if tubes held 3–24 h at RT or 4°C for 3–24 h^70^ Plasma Aβ_42_ and Aβ_40_ stable up to 24 h at 4°C (but not at RT) and decrease afterwards; effects can be mitigated by use of Aβ_42/40_ ratio in most assays*^70^ Short term storage at <24 h had no effect on any markers except for total tau which decreased if stored for ≥2 weeks at 4°C^70^ Fresh EDTA plasma storage +4°C; time to measurement (h): (0, 1, 3, 6, 24) stable up to 6 h (Roche-Elecsys)^73^ Aβ_42_ and Aβ_40_ decreased after intermittent ≤2-week storage at 4°C before −80°C*^70^ No effect of intermittent ≤2-week storage at −20°C (before −80°C) for any markers^70^
EDTA plasma storage after thawing	+4°C; time to measurement (h): 0, 1, 6, 24	Aβ_42_ and Aβ_40_ stable up to 6 h at +4°C and 1 h at RT (Roche-Elecsys)^73^
	RT; time to measurement (h): 0, 1, 6, 24	Aβ_42/40_ ratio stable up to 24 h (both +4°C and RT) (Roche-Elecsys)^73^
Number of tube transfers	≤5	No effect^73^
Effect of the collection tube on stability during freezing	Material (PP, PET)	No effect^70,73^
	Sample size (mL): 2.7, 4.9, 8.5, 9, 10	No effect^73^
	EDTA cation (K2, K3)	No effect^73^
	Gel separator (+/-)	No effect^73^
Freeze/thaw cycles (from −80°C)	≤ 4 cycles	No effect on serum NfL, plasma Aβ_42_, total tau levels by Simoa^74^ Minor reductions in plasma Aβ_40_ (Simoa) at cycle 3 with further reductions with ≥ 4 cycles^74^ No effect on plasma Aβ_42_ and Aβ_40_ levels (various assays)*, p-tau_181_, tau, or NfL^70^ GFAP levels increased after ≥4 cycles^70^ Decrease in plasma p-tau_181_ at ≥4 cycles, decrease in plasma EDTA Aβ_40_ at ≥4 cycles can be mitigated by Aβ_42/40_ ratio^71^ Serum NfL and GFAP stable up to 4 cycles, serum Aβ_42_ and Aβ_40_ and total tau reduced after 2 cycles^71^
Freeze/thaw cycles (−25°C)	≤3 at −20°C or −25°C	No effect on Aβ_42_, Aβ_40_, tau, or p-tau_181_^73^
Circadian rhythm	Time of day of blood collection: 8:00, 12:00, 15:00 >24 h	Small effect on Aβ_42_, Aβ_40_ and tau, no effect on Aβ_42/40_ ratio (Roche-Elecsys)^73^ No diurnal variation in plasma Aβ levels (INNO-BIA)^72^

*Various assays including Precivity-AD (C2N Diagnostics) which is immunoprecipitation liquid mass spectrometry, a MALD-TOF-MS method using a protocol modified from Nakamura et al.,^76^ Euroimmun ELISA, Aracion ELISA, Simoa 4-PLEX E, and Simoa 3-PLEX A.

OAβ: Aβ oligomerization tendency assay

## Neuropsychiatric symptoms overview

Evaluating biomarker levels in relation to NPS requires an accurate definition of what constitute NPS. Most definitions agree that NPS must emerge in the context of an underlying neurological disease, affect function beyond what can be attributed solely to the disease, and persist for a minimum time frame at a specified severity level.^77–79^ In addition, other potential causes (e.g., delirium or psychiatric conditions unrelated to NDD) must be excluded as the primary cause of NPS, although “delirium-like” NPS have been defined as a possible prodrome of dementia with Lewy bodies (DLB).^80^

### Mild behavioral impairment

Over time, the definition of NPS in dementia has become more nuanced due to several advancements in the field. First, there is growing recognition that NPS may emerge in advance of dementia, and sometimes even in advance of cognitive decline, as opposed to being limited to moderate to severe dementia. For example, the construct of mild behavioral impairment (MBI)^79^ stipulates that NPS may occur well before clinical dementia during the preclinical or prodromal disease phases. MBI symptoms comprise the domains of decreased drive and motivation (apathy), emotional dysregulation (mood/anxiety), impulse dyscontrol (impulsivity, agitation, abnormal salience), social inappropriateness (impaired social behavior), and abnormal perception or thought content (psychosis). Early in the disease process, however, MBI can present as a variable or subtle mix of symptoms from several domains. Thus, assessing the global construct of MBI may offer advantages for early detection and prognostication over individual domains.^81,82^

### Syndromic criteria for NPS in neurodegenerative diseases

Another advancement in NPS research pertains to the development or refinement of syndromic diagnostic criteria for NDD-related behavioral changes (i.e., apathy, psychosis, agitation) as opposed to simple consideration of individual symptoms with variable measurement approaches. For example, apathy criteria developed by Alzheimer's Association International Society to Advance Alzheimer's Research and Treatment (ISTAART) and International Society for CNS Clinical Trials Methodology working groups, using a modified Delphi process and expert consensus, specify: 1) the presence of NDD; 2) symptom presence for a minimum of four weeks; 3) a change from the individual's baseline; 4) symptoms in two of the following dimensions: diminished initiative, interest, and emotional expression/responsiveness; and 5) exclusion of other confounding factors.^83^ Similarly, revised criteria for AD-related psychosis developed by an expert panel led by the International Psychogeriatric Association (IPA),^84^ and the research framework developed by ISTAART^85^ emphasize that: (1) symptoms may emerge in MCI (for IPA criteria) or even in SCD or CU stages (for ISTAART criteria); (2) symptoms must be present for a minimum of four weeks; (3) overlap with agitation and affective symptoms must be noted (IPA) or are exclusionary if psychotic symptoms are better accounted for by an affective or agitation syndrome (ISTAART); (4) symptoms may be fluctuating or persistent; (5) symptoms must affect function; and (6) other etiological categories must be excluded. The syndromic criteria for agitation, developed by an IPA working group, specify: (1) the existence of dementia or a pre-dementia syndrome (i.e., MCI); (2) behaviors in one or more of verbal aggression, physical aggression, and excessive motor activity domains that are associated with distress and impaired function; and (3) not attributable solely to another medical or psychiatric etiology.^78^

While revisions to existing criteria have helped clarify distinct phenotypic NPS presentations in the context of NDD, other behaviors that overlap with primary psychiatric disorders, such as hoarding, obsessive-compulsive disorder, disinhibition, impulsiveness, and affective dysregulation, remain understudied. A key question remaining in the characterization of NPS is the extent to which these symptoms can be differentiated from pre-existing or primary psychiatric pathology, currently better determined by symptom natural history. This critical area needs biomarkers, particularly minimally invasive biofluid markers that can be readily translated to various clinical and research settings. Ultimately, these biomarkers would help distinguish primary psychiatric symptoms from NPS related to NDD and also determine if there are comorbid primary psychiatric–NDD etiologies. Depression, for example, may reflect recurrent early-onset depression, late-life depression, or late-onset depression as a manifestation of NDD, with few cross-sectional clinical features clearly distinguishing one from the other. Similarly, late-life schizophrenia may be hard to distinguish from NDD-related psychosis, although symptom phenomenology might help distinguish the underpinning pathology.^86^ For example, the presence of complex delusions and auditory hallucinations would more strongly indicate late-life schizophrenia, whereas visual hallucinations and misidentification delusions may suggest prodromal DLB, and delusions of theft are the most common psychotic symptom in AD.^84^^87–89^ However, the identification and utilization of biofluid biomarkers could be fundamental for differentiation when psychosis phenomenology is not so distinct and symptoms related to both clinical entities coexist.

An important issue concerning NPS is the extent to which symptoms may overlap, co-occur, or fluctuate between assessments. Factor analyses suggest that symptoms can be subdivided into separate subgroups, such as depression, agitation, psychosis, and elation.^90^ While it is generally acknowledged that certain symptoms may co-exist with each other, few biomarker studies to date have addressed this issue by focusing on subtypes or by controlling for the presence of specific NPS when examining individual symptoms.

Most biomarker studies published to date examining correlates of NPS have relied on established scales such as the Neuropsychiatric Inventory (NPI).^91,92^ The NPI quantifies NPS across 12 domains: delusions, hallucinations, agitation/aggression, dysphoria, anxiety, euphoria, apathy, disinhibition, irritability/lability, aberrant motor activity, night-time behavioral disturbances, and appetite/eating abnormalities. Severity scores for each domain are rated 1 to 3, where 1 is the lowest severity and 3 is the highest. An additional score out of 5 is given to rate caregiver burden, with 1 being the lowest and 5 being the highest. Most research has used the NPI, which has demonstrated excellent utility. In the ADMET 2 clinical trial of methylphenidate for apathy, for example, a score of ≥4 to identify clinically significant apathy corresponded well with apathy diagnostic criteria.^93^ Other scales used in biomarker studies, particularly those in preclinical and prodromal samples of older adults, include symptom-specific assessments focused on traditional psychiatric phenomenology (e.g., Patient Health Questionaire-9,^94^ Geriatric Depression Scale,^95^ Beck Anxiety Inventory^96^).

More recently, NPS in patients with preclinical or prodromal NDD, predominantly AD but also Parkinson's disease/Lewy body diseases, have been assessed using the Mild Behavioral Impairment Checklist (MBI-C), which evaluates the presence and severity of five MBI symptom domains, consistent with ISTAART MBI diagnostic criteria: decreased motivation, emotional dysregulation, impulse dyscontrol, social inappropriateness, and abnormal perception or thought content.^81,97^ The MBI-C specifies that these NPS must be later-life emergent, persist for at least six months, and represent a change from the person's baseline. Although the MBI-C was initially developed for the assessment of MBI in older adult populations without dementia, it is still a valid tool to assess NPS in older adults living with dementia.^98^ When the MBI-C is not available, for instance, in legacy datasets, MBI can still be operationalized and studied in relation to blood biomarkers using the NPI and its derivatives.^99^

## Importance of specifying cognitive status when reporting neuropsychiatric symptoms

NPS are prevalent across the spectrum of neurocognitive disorders, from CU through MCI and all stages of dementia.^100,101^ While the range of NPS is broadly similar, comprising changes in apathy, mood/affect, agitation, disinhibition, and psychosis, the rationale underlying the search for biomarker correlates will likely differ depending on the cognitive status. Studies exploring the association between NPS and cognition using a nomothetic approach have generally shown that the presence of NPS is either consistent or increases as cognition worsens and disease progresses.^102^ Consistently, several studies have found greater NPS frequency with worse cognitive status and/or progression across the AD continuum (i.e., controls, SCD, MCI, dementia).^103,104^ Meta-analytic evidence further supports significant associations between NPS and cognition, as well as a greater presence of NPS in patients with dementia who have poorer cognitive performance.^105^

Additionally, studies have suggested that NPS, particularly those meeting MBI criteria, in participants with MCI are associated with both a higher rate of progression to dementia^99,106^ and a lower probability of reversion to CU,^107^ regardless of the presence of Aβ pathology.^108^ In addition to the potential mediating role of cognition in the association between biomarkers and NPS,^109^ other factors, such as sex, cognitive complaints, worries, ethnicity, and apolipoprotein E gene (*APOE*) haplotypes, seem to modulate the strength of the association between NPS and AD biomarkers or incident dementia.^103^^110–114^

While evidence has strongly linked MBI to elevated dementia risk,^99,11^^,^^21,15–120^ there remains a degree of uncertainty as to whether later-life emergent NPS are sequelae of underlying NDD or psychiatric risk factors for cognitive decline and dementia.^115,121^ Notably, the hazard of incident dementia associated with MBI differs based on cognitive status. For example, when comparing MBI-psychosis to no NPS, the hazard ratio (HR) for incident dementia was 9.96 in CU and 3.38 in MCI.^122^ While MBI-psychosis was more frequent in MCI, with a higher absolute progression rate to dementia than in CU, these findings suggest that the earlier MBI emerges, the more significant its relative contribution to risk. Similar findings have been reported for MBI domains of decreased motivation and emotional dysregulation.^111,112^ Thus, while incorporating natural history and cognitive status is helpful in classifying NPS, biomarkers can help further differentiate NPS due to NDD, normal aging, or other psychiatric conditions in both clinical and research settings, especially in CU and SCD populations. Molecular differences, general and sex-specific, may contribute to the heterogeneity of NPS in different neuropsychiatric conditions, potential targets for multiomic approaches aiming at the development of precision medicine strategies.^123^ In this respect, biomarkers for behavior are analogous to biomarkers for cognition, namely, to differentiate normal cognitive aging from pathological changes due to underlying NDD, which can be challenging in otherwise healthy individuals.^124^

## Data and results so far on studies of neuropsychiatric symptoms and plasma biomarkers across the cognitive continuum

Biomarkers specific to NPS may differ from biomarkers of NDD, as previously demonstrated in CSF.^125^ NPS may arise due to myriad possible etiologies, influenced in part by the nosology and nomenclature applied in detection and measurement. NPS biomarkers may guide symptom-specific treatment and can be used in conjunction with disease biomarkers to identify the underlying etiology. Presently, much more work is needed to understand when and how to differentiate late-life NPS due to various possible etiologies, but the application of AD blood biomarkers is a logical starting point. Here, we summarize data published so far on the association between NPS and plasma biomarkers. Recognizing the importance of specifying cognitive status when discussing NPS, we stratify findings based on cognitive group (i.e., CU, SCD, MCI, dementia) where possible, acknowledging that some studies were also conducted in mixed samples with various cognitive statuses.

### Cognitively unimpaired adults

Several studies have reported significant cross-sectional and longitudinal associations between Aβ pathology and at least some NPS in CU participants. A review of these studies has been published elsewhere.^28^ Cross-sectional, positive associations were found between Aβ deposition and anxiety and MBI-C total scores in CU participants.^126,127^ Longitudinal evidence has shown that: (1) depressive^128^ and anxious^129^ symptoms in participants without objective cognitive impairment successfully predicted cognitive decline, particularly in the presence of Aβ deposition; and (2) Aβ pathology at baseline was significantly associated with longitudinal increases in depression and anxiety symptomatology in CU participants.^130–132^

The association between tau burden and NPS has been less studied, and the results are less conclusive. The bulk of the evidence arises from established biomarker approaches, and major evidence in favor of significant associations comes from longitudinal studies of CU participants at baseline rather than cross-sectional designs.^28^ One study reported positive correlations between baseline CSF total tau and p-tau_181_ levels and longitudinal increases in NPI-Q scores and mood/affective symptoms.^130^ Another study observed significant associations between neuritic plaques and psychotic symptoms (but not NPI-Q total scores).^133^ A recent longitudinal study of 286 CU older adults at baseline found that NPS using the NPI-Q were independently associated with greater progression to MCI over an eight-year period (while adjusting for CSF and PET biomarkers).^134^ For MBI, in a large sample of CU participants, global MBI status was found to predict clinically diagnosed and neuropathologically confirmed AD.^119^ This finding was later replicated in a cohort consisting of both CU and MCI participants.^120^ Psychosis, while low frequency, had the strongest effect of all MBI domains. Another large study of CU and SCD participants found that in reference to a no NPS group, CU participants with MBI had a 2.76-fold greater incidence of dementia and SCD participants with MBI had a 1.99-fold greater incidence of dementia. For conventionally measured NPS not meeting MBI criteria, incidence rates did not differ from the no NPS group. Of MBI progressors to dementia, 76% developed AD.^135^

A small study that included CSF biomarkers provides biological insights into NPS in preclinical AD. In Aβ-positive participants who were CU with or without SCD, NPS measures (i.e., MBI-C) were associated with early-stage AD Braak region tau-PET tracer binding and higher CSF p-tau_181_ levels, while cognitive performance measures (i.e., ADAS-DR) were not linked to tau.^136^

### Dementia-free older individuals (mixed CU, SCD, and MCI)

Several studies have explored the association between NPS and blood-based biomarkers in mixed samples that include CU, SCD, and MCI, sometimes denoted “dementia-free”. In a 5-year study of 331 dementia-free older adults (47 with MCI), higher plasma Aβ_42_ levels at baseline predicted incident late-onset depression.^137^ In another 6-year study of 223 dementia-free individuals, those with depression and lower Aβ_42/40_ at baseline were at greater risk of incident AD compared to those with non-Aβ depression.^138^ Of those with Aβ-associated depression, *APOE* ε4 carriers were at greater risk of AD than non-carriers.

Some studies also investigated associations between blood-based non-core AD biomarkers and individual NPS, mainly depression. One study used targeted proteomics in ADNI to explore links with depressive symptoms. Here, hepatocyte growth factor, insulin polypeptides, pregnancy-associated plasma protein-A, and vascular endothelial growth factor were found to be related to depression.^139^ Other than depression, one cross-sectional aging study found that lower vitamin B_6_ levels were associated with anxiety.^140^ These studies exploring the relationship between blood analytes, using various assays with unknown levels of precision to NPS symptoms, and with limited or no replication to date serve to highlight the wide range of possibilities to be explored in future research.

The MBI construct has been particularly useful for better understanding the relationship between NPS and core AD plasma biomarkers amongst dementia-free older adults, highlighting the importance of incorporating natural history into NPS assessment. To date, several longitudinal studies have been published on this topic. MBI was shown to be associated with higher plasma p-tau_181_ levels over 4 years, an association not seen in participants with NPS that did not meet MBI criteria (i.e., non-MBI NPS).^141^ In addition to a decline in memory and executive function, survival analyses demonstrated a 3.92-fold greater dementia incidence in MBI participants, with no significant differences between the non-MBI NPS and no NPS groups. Recently, a secondary analysis of data from a nutritional supplement randomized controlled trial was reported, in which both MBI and plasma p-tau_181_ were measured.^142^ In this sample of dementia-free older persons (60% MCI), MBI-psychosis was associated with an increase in plasma p-tau_181_ over one year. Further, a tau-PET study demonstrated effect modification by amyloid status for MBI-associated tau tracer uptake in early-stage AD Braak regions; the association was observed only in Aβ-positive participants.^143^ These findings extend those from the BioFINDER-2 preclinical-AD study,^136^ and highlight a need to investigate the relationship between MBI and plasma tau in the context of Aβ. Most recently, in a study of 101 dementia-free older adults, those with MBI had higher plasma p-tau217 levels and higher odds of p-tau217 positivity than participants without MBI.^144^ These findings support the growing evidence base linking MBI with AD proteinopathies, and the utility of MBI status for disease detection in advance of dementia.

Aside from studies focusing solely on plasma tau biomarkers, a cross-sectional study of 139 dementia-free participants (53 with MCI) reported an association between lower plasma Aβ_42/40_ and greater MBI burden. Among MBI domains of affective dysregulation, decreased motivation, and impulse dyscontrol, only affective dysregulation was significantly associated with lower plasma Aβ_42/40_.^145^ In a longitudinal study of 583 dementia-free older adults (330 with MCI), MBI was associated with a greater increase in plasma NfL levels.^146^ Although not in plasma, it should be noted that MBI participants exhibited CSF p-tau_181_, p-tau_181_/Aβ_42_ ratio, and tau/Aβ_42_ ratio trajectories consistent with NDD across two independent cohorts with MCI; in one cohort, MBI was also related to Aβ_42_ and Aβ_42/40_ changes over 4 years. In contrast, these same biomarkers did not differ between participants with non-MBI NPS and no NPS, except for a longitudinal association with total tau in one cohort. Importantly, of MBI progressors to dementia, 81% developed AD.^147^

There have been some studies investigating the association between other plasma analytes and NPS in non-dementia samples. One study has shown that the presence of inflammatory cytokine interleukin-(IL)-1β correlates with agitation severity.^148^ Higher plasma levels of IL-6 were also associated with a higher prevalence of depression in a cohort of 69 CU or MCI individuals.^149^ Monocyte chemoattractant protein-1 (MCP-1) was associated with future risk of depression.^150^ One cross-sectional aging study found that lower vitamin B_6_ levels were associated with anxiety.^140^

### Mixed samples including dementia

Comparatively few studies have combined participants with and without dementia into one study cohort. One cross-sectional study of 1005 older adults (118 with MCI or dementia) found an association between plasma p-tau_181_ and p-tau_217_ and greater agitation and disinhibition.^151^ A study in a memory clinic cohort including CU older participants and patients with MCI and mild dementia found no association of plasma p-tau_181_, GFAP, and NfL with overall NPS cross-sectionally, but GFAP and NfL were associated with greater NPI-Q scores over time.^152^ Applying an untargeted proteomics approach in the same cohort revealed that a combination of 15 plasma proteins, including C-reactive-protein (CRP), complement factor H, alpha-1 microglobulin, noelin, and apolipoprotein H, predicted current and future NPS,^153^ which was further validated in the ADNI cohort.^154^ Finally, a study of patients from outpatient memory clinics found a correlation between brain-derived neurotrophic factor (BDNF) levels and aggressiveness.^155^

A multiplex biochemical biomarker study of 146 known plasma analytes from the Alzheimer's Disease Neuroimaging Initiative (ADNI) found several analytes that were highly associated with depressive symptoms, including hepatocyte growth factor, insulin polypeptides, pregnancy-associated plasma protein-A, and vascular endothelial growth factor.^139^ Medications, body mass index, *APOE* alleles, serum glucose, and CSF Aβ levels were also assessed and did not significantly affect the associations between these plasma marker levels and depressive symptom scores. Future studies in this area could conduct subgroup analyses examining the levels of these plasma analytes comparing MCI, probable AD groups, and CU individuals.

A few studies have also explored the associations of NPS with plasma inflammatory biomarkers in samples with mixed cognitive statuses. A memory clinic cohort found that NPS were associated with a panel of serum inflammatory markers, including IL-6, eotaxin-3, and CRP.^156^ In a small sample of 27 individuals with mild AD or amnestic MCI, those with more apathy symptoms had higher serum levels of soluble tumor necrosis factor (TNF) receptors.^157^ Nevertheless, it should be taken into account that these blood-based inflammatory markers are poor and non-specific biomarkers of clinical findings in general.^158^ Given the range of analytes studied and assays used, much work remains to be done to establish which, if any, non-core AD biomarkers reliably predict emergence of specific NPS symptoms.

### AD dementia

AD, the most common type of dementia, is very frequently accompanied by NPS, including apathy, motor disturbances, depression, anxiety, agitation and, less commonly, delusions, hallucinations, and disinhibition.^26^ Owing to its high prevalence, a large amount of research has been conducted on NPS in AD dementia relative to preclinical or prodromal stages of NDD. The wealth of research in this area has enabled a more precise understanding of individual NPS in relation to plasma biomarkers, as summarized below.

#### Agitation

In recent years, agitation in patients with AD has been associated with several blood-based biomarkers. As observed in non-dementia samples, high levels of IL-6, cortisol, and greater natural killer cell activity, all indicating pro-inflammatory conditions, were associated with agitation severity.^159^ However, there is conflicting evidence regarding the relationship between IL-6 and agitation, with one study finding no association in their sample.^160^ Baseline TNF levels^160^ and 4-hydroxynonenal^161^ have been both observed to be associated with agitation severity and oxidative stress.

In AD carriers of the *APOE* ε4 allele, plasma Aβ_42/40_ has been associated with longitudinal increases in agitation.^162^ Elevated levels of irisin have been reported in patients with agitation and were positively correlated with the duration of agitated symptoms.^163^ While BDNF was associated with agitation in a mixed cognition cohort, as previously mentioned,^155^ this association did not hold in samples comprising only AD dementia participants.^163^ In females, the *APOE* ε4 allele confers the greatest risk of agitation severity compared to other allele combinations.^164^ While there have been various primary investigations assessing the relationships between blood biomarkers and agitation severity, many of the studies have not yet been replicated and sometimes report conflicting results.

#### Apathy

Apathy has been observed in some cohorts to be the most prevalent NPS in AD, with a point prevalence of 49%.^165^ Positive correlations between apathy and plasma levels of γ-aminobutyric acid (GABA), a key inhibitory neurotransmitter in the central nervous system, have been observed.^166^ The presence of apathy has also been associated with a higher plasma/platelet clusterin ratio in AD.^167^ However, evidence for the association between plasma homocysteine levels and apathy has been mixed. In one study, plasma homocysteine levels were associated with total Apathy Evaluation Scale (AES) scores in a sex-dependent manner: females with apathy had significantly higher homocysteine levels than those without. After controlling for cognition, depression, and vascular risk factors, plasma homocysteine was correlated with the cognitive and behavioral subdomains of AES, but not with the emotional domain.^168^ In contrast, another group found no significant difference in mean plasma homocysteine levels between patients with AD who had NPS and those who did not.^169^ One explanation for this discrepancy could be the way NPS were operationalized as well as possible differences in assays; in the second study, apathy was included within an ‘affective cluster’ alongside anxiety, depression, and irritability, potentially obscuring distinct associations with apathy specifically.^169^

#### Depression

In studies examining depression, positive correlations were found between plasma GABA levels and depression scores on the NPI.^166^ Another study reported differing plasma homocysteine levels in patients in the moderate AD stage with or without depression; plasma homocysteine levels were higher in patients with major depression compared to those without major depression.^170^ Moreover, patients with moderate AD also exhibited more severe behavioral disturbance symptoms on the Cornell Scale for Depression in Dementia than patients with mild AD, suggesting a potential relationship between elevated homocysteine levels, more burdensome behavioral disturbances associated with major depression, and dementia progression.

Dementia stage-specific associations between plasma ceramide levels and NPS were also observed in another study of patients with mild and moderate-to-severe AD; specifically, plasma ceramide levels were positively associated with NPI-depression only in those with moderate-to-severe AD.^171^ However, another study reported elevated plasma ceramide levels in patients with recent major depression (within two years) compared to patients with past or no history of major depression, regardless of dementia status.^172^ A major limitation of this latter finding was the small sample size and the combination of CU individuals with patients with AD, as no significant differences in mean ceramide levels were found between these two groups.

One study examined plasma cortisol concentration and depressive symptoms.^173^ Although mean plasma cortisol was higher in AD compared to controls, it was only slightly higher in patients with AD who had depression than in those who did not have depression. This study concluded that plasma cortisol levels were more reflective of cognitive impairment than the severity of depression in this population.

#### Psychosis

Several blood-based biomarkers have been assessed for their relationship with the severity of psychosis among patients with AD. Homocysteine serum levels were positively correlated with psychotic symptom severity,^174^ whereas IL-7, IL-15, and IL-18 were all negatively correlated with psychosis severity.^175^ When stratified by *APOE* ε4 carrier status, IL-15 was the best predictor of total NPS (including psychosis) in carriers, while IL-18 and TNF were the best predictors among non-carriers.^176^ Many of these biomarker assessments are standalone investigations that require replication to improve the evidence supporting the utility of these biomarkers for patient risk assessments and treatment response observations in the context of psychosis.

#### Less common NPS in AD

Plasma/platelet clusterin ratio has been observed to be associated with greater severity in NPI domains of disinhibition, aberrant motor behavior, and irritability in patients with AD.^167^ Another group found positive associations between plasma ceramides and delusions in the mild AD stage.^171^ Other NPS, such as hallucinations, elation, anxiety, and appetite changes, are less frequent and did not have positive findings in other studies. Many of the studies focusing on less frequent NPS reported associations with CSF biomarkers, not plasma, and hence were not included in this review.

#### NPS in AD genetic association studies

Analysis of plasma samples has facilitated the study of genetic factors and their role in developing NPS in AD, sometimes implicating certain pathogenic pathways or specific neurotransmitter systems. The ε4 allele of the *APOE* gene is widely recognized as the most substantial genetic risk factor for sporadic late-onset AD.^177^ A genome-wide association study identified a significant association between the *APOE* ε4 allele and psychosis in AD.^178^ However, a review paper on *APOE* haplotypes and NPS in AD revealed inconsistent findings: some studies found significant associations between the *APOE* ε4 allele and more agitation/aggression, hallucinations, delusions, and late-life depression or anxiety, while other studies failed to find any association.^179^ A systematic review and meta-analysis of 53 studies found no association between *APOE* ε4 alleles and depression, anxiety, apathy, agitation, irritability, or sleep disturbances in MCI or AD.^180^ However, it has been proposed that *APOE* ε4 alleles may differentially exert their effects on behavioral symptoms in AD depending on the cognitive stage^181^ or may act as modulators of the effects of other genes on such behavioral manifestations,^182^ particularly in earlier dementia stages.^183^ Recent studies on sex-specific associations found that *APOE* ε4/ɛ4 females showed increased NPS burden and higher NPS severity scores than *APOE* ɛ3/ɛ3 females, with no such association in males.^164^ A 1-year longitudinal study of 793 patients with AD found that participants with the *APOE* ε4 allele exhibited symptoms of irritability/lability, delusions, hallucinations, and agitation/aggression.^184^

The remaining studies reviewed here are candidate gene association studies which are prone to false positives so the findings should be interpreted with caution. Dopamine function is mediated by five distinct receptor subtypes (DRD1, DRD2, DRD3, DRD4, and DRD5).^185^ Studies have been conducted to examine the associations of NPS among patients with AD with dopamine receptor polymorphisms. One study found that psychosis and aggression were significantly more prevalent in *DRD1* B2/B2 homozygotes, while psychosis alone was more prevalent in *DRD3* B1/B1 or B2/B2 homozygotes.^186^ Neither aggression nor psychosis were associated with variants of the *DRD2* or *DRD4* genes.^186^ Similarly, another study found associations between *DRD1* variants and psychosis and aggressive behavior, as well as an association between a *DRD3* polymorphism and psychosis, but not with aggression in AD.^187^ Specifically, carriers of the *DRD1* B2 allele displayed more frequent symptoms of aggression and hallucinations, and individuals homozygous for the *DRD3* B1 allele were more likely to experience delusions. These findings contrasted with those of a previous study where homozygosity for either *DRD3* allele was associated with psychosis in AD.^186^ Another study, however, did not confirm these associations between *DRD3* polymorphisms and psychotic symptoms (delusions and hallucinations) on the NPI.^188^ A longitudinal study of 395 patients with probable AD found associations between *DRD3* and elation, and between *DRD4* with agitation/aggression and *DRD4* with depression; however, these findings did not remain significant after correction for multiple testing.^189^ *DRD1* and *DRD2* were not associated with these symptoms, and no significant associations were found between *DRD1-4* variants and the other NPS of delusions, hallucinations, psychosis, and aberrant motor behavior. To conclude, larger studies and longitudinal data are required to better understand the relationship between *DRD1-4* variants and NPS.

Serotonergic function is modulated by the serotonin transporter (SERT), which is responsible for the reuptake of 5-hydroxytryptamine (5-HT) at the synapse. SERT is an important target for the pharmacological management of psychiatric symptoms. Two variants that influence the transcription rate of the *SERT* gene are the linked polymorphic region (LPR) variant and the variable number tandem repeat (VNTR) variant. One study found the 102 T polymorphism of the 5-HT2A receptor to be significantly associated with delusions and agitation/aggression in 96 patients with AD,^190^ consistent with other studies.^191^ However, one study showed no associations between LPR and symptoms of depression, psychosis, anxiety, or agitation.^192^ A longitudinal study of 367 patients with AD found a significant relationship between the LPR variant long allele and NPI-irritability scores, and between the VNTR variant 10-repeat allele and NPI-psychosis scores. No associations between these variants were found with depression, anxiety, or agitation/aggression.^192^ To conclude, SERT could play a role in the development of symptoms of irritability or psychosis, where these findings could be replicated further in larger cohorts. Associations with anxiety and depressive symptoms could also be further studied in relation to the *SERT* gene.

Various genetic markers have been implicated in psychotic symptoms. The rs2153674 single nucleotide polymorphism of the G72 locus, implicated in other psychotic disorders, was significantly correlated with the severity of delusional aspects of AD-induced psychosis.^193^ In a single nucleotide polymorphism investigation, the T allele of rs6494223 of the *CHRNA7* gene (the gene for the alpha 7 nicotinic acetylcholine receptor) was associated with a higher severity of delusions, with cognition being controlled for using the Mini-Mental State Examination.^194^

### Vascular cognitive impairment and dementia

Vascular cognitive impairment and dementia (VCID) refers to cognitive decline in which cerebrovascular disease either causes or contributes to the cognitive syndrome.^195^ Diverse pathophysiological mechanisms may underlie VCID, including cerebral small vessel disease, ischemic stroke, macro- and micro-hemorrhages, and CAA.^196^ Traditionally, neuroimaging has been central to the identification of cerebrovascular pathology and the pathophysiological processes that lead to VCID.

Compared to core AD biomarkers, blood-based biomarkers for VCID are less established. CAA is one potential cause of VCID, leading to both small vessel disease (e.g., lacunar infarctions, periventricular hyperintensities, cerebral microhemorrhages) and macrohemorrhages. CAA has been associated with lower Aβ_40_ measured in the CSF.^197^ However, only lower Aβ_42_ in the hereditary Dutch-type CAA was found to be significant, while no differences were observed between sporadic CAA and control groups for Aβ_38_, Aβ_40_, or Aβ_42_.^198^ Emerging blood-based biomarkers for VCID due to subcortical small vessel disease include inflammatory cytokines, markers of oxidative stress, and endothelial-derived adhesion proteins (e.g., soluble intracellular adhesion molecule-1 and soluble vascular cell adhesion molecule-1).^199,200^

Depression is the best-studied NPS in VCID, and literature on ‘vascular depression’ may be sufficiently salient to inform future work on blood-based biomarkers of NPS in VCID.^201^ While not studied specifically in those with VCID, older adults with depression have been found to have a greater burden of white matter hyperintensities on neuroimaging as well as higher levels of IL-1β and TNF receptor-2, and lower levels of glial-derived neurotrophic factor, adiponectin, and serum brain-derived neurotrophic factor.^201^ The recent identification of brain endothelial injury markers, such as vascular-endothelial cadherin, and their association with AD pathology and cognitive outcomes in preclinical AD, offers opportunities to further explore associations of their plasma levels with NPS in AD.^202^

### Frontotemporal dementia

FTD encompasses a class of NDDs characterized by progressive neuronal loss in the frontal and temporal lobes, with a typical onset in middle age.^203^ While there is heterogeneity in clinical presentation, FTD is most often characterized by language and communication impairments (e.g., primary progressive aphasia [PPA]) and/or abnormal behavior.^204^ Behavioral variant FTD (bvFTD) is typified by disinhibition, personality changes, and a decline in socially accepted behavior, judgment, and self-control. These often co-occur with other NPS, of which the patient is typically unaware. The relationship between plasma biomarkers and NPS in FTD is an area of active research.

NfL, a measure of neuro-axonal damage, has been a prime candidate for discriminating FTD from AD in both CSF and plasma studies, with comparable findings.^205^ NfL has also demonstrated high discriminatory power (area under the receiver operating characteristic curve of 0.84 to 0.96) to differentiate bvFTD from primary psychiatric disorders (e.g., major depressive disorder, schizophrenia, conversion disorder).^206,207^ Recent work has found that plasma GFAP, a measure of astrogliosis, was elevated in patients with FTD (n = 72) compared to those with AD (n = 56); there were no group differences in NfL.^208^ Plasma GFAP was also associated with progression to moderate FTD. Additionally, plasma GFAP was significantly higher in patients with FTD with symptomatic *GRN* pathogenic variants compared to *C9orf72* expansion carriers or patients with *MAPT* pathogenic variants.^209^ Finally, plasma tau concentrations were found to be higher in bvFTD (n = 71) and PPA (n = 83) compared to healthy controls (n = 22), as well as in patients with *MAPT* pathogenic variants compared to those with *C9orf72* expansions or *GRN* pathogenic variants.^210^

FTD can be challenging to diagnose, especially at its early stages, as its NPS often overlap with symptoms due to other etiologies. Given this overlap, most biofluid studies of NPS in FTD examine differentiating dementia etiologies and time to cognitive impairment.^12^ Biomarkers help to understand the biological underpinnings of these symptoms. The levels of certain biomarkers might be associated with the degree of neuroinflammation or neuronal loss in specific brain regions, which in turn could relate to the severity of NPS in dementia.

### Dementia with Lewy bodies

DLB is the second most common neurodegenerative type of dementia in older people, resulting from the aggregation of α-synuclein.^211^ Core features of DLB include visual hallucinations and REM sleep behavior disorder (RBD), though other behavioral symptoms, including depression, apathy, anxiety, hallucinations in other sensory modalities, and delusions, may also occur.^212,213^ Together, these symptoms often cause greater behavioral burden than in other dementia syndromes.^214,215^ Proposed biomarkers for DLB encompass various modalities such as single-photon emission computed tomography (SPECT), magnetic resonance imaging (MRI), polysomnography, CSF analysis, PET, and skin biopsies.^213^ While there are many tools available to help assist with the detection of the various biomarkers and symptoms associated with DLB, there is very little research to date on plasma biomarkers that could be utilized to detect DLB or the behavioral symptoms that may accompany Lewy body diseases. A study on the association between NPS and neuropathological correlates suggests that LB and AD pathology contribute differently to NPS, likely through an additive process.^216^ However, studies involving biomarkers to confirm these findings are still lacking. The few studies that have investigated plasma biomarkers in the context of DLB have done so in relation to cognitive performance and isolated RBD,^217,218^ or have mixed samples including older adults with other NDDs.^219^ The dearth of research investigating the relationship between these symptoms and pathological changes highlights the critical need for more research in this field.

## Ethical aspects of biomarker research in neuropsychiatric syndromes

AD biomarker testing may be performed by research studies, clinical trials, or as part of a clinical evaluation.^220–222^ In all settings, individuals should be offered a choice of whether or not to learn their results,^223–225^ a practice that supports commitment to patient-centered care, transparency, and informed patient choice. Effective communication and support during the process of sharing biomarker information with patients is important because learning about risk can be distressing. Studies demonstrate that patients do experience distress regarding the return of biomarker results.^226^ Individuals watching amyloid PET disclosure videos experience declines in positive feelings and increases in negative feelings.^227^ Best practices for disclosure to promote autonomy and well-being include pre-test counseling and post-test explanations, guidance, and support, including therapeutic alternatives and information on healthy life choices.^228^ A comprehensive example is the Biomarker Disclosure Toolkit.^229^

With the advent of the U.S. Food and Drug Administration-approved AD-specific treatments, it is important to promptly identify potential candidates for treatment (i.e., patients with a clinical diagnosis of early symptomatic AD with biomarker confirmation of Aβ pathology). While there are many reasons why clinicians may not provide a clear diagnosis,^230–235^ these delays increase the risk of NPS, which typically worsen as dementia progresses and complicate collaborative decision-making. Patients may be less likely to recognize their symptoms due to anosognosia.^236^ NPS, such as apathy, anhedonia, impulsivity, and delusions, can exacerbate existing issues with the ability of patients to protect vital self-interests such as financial security.^237^ Delays thus disempower patient choice, especially since certain treatments are likely to be most effective early in the disease.^238^

The field of AD biomarkers is rapidly evolving, and the appropriate use of biomarkers depends on clinicians being knowledgeable about recent developments. For example, with their greater accessibility compared to CSF testing or imaging techniques, blood-based biomarkers may expand access to AD biomarkers in socioeconomically disadvantaged societies, including low- and middle-income countries.^239^ However, most AD biomarker studies have been performed in cohorts lacking racial and ethnic diversity that are healthier, have higher education levels, and have higher incomes than the general population.^240,241^ This is especially concerning because biomarker values appear to be associated with factors such as race, medical conditions, and greater contextual factors that encapsulate structural and social determinants of health.^55^^242–244^ Although using different cut-offs for different groups has been proposed, it is vital to avoid adjusting for group differences in a manner that perpetuates disparities.^245^ For example, it would be preferable to use biomarker measures that perform consistently across groups or to adjust for factors (e.g., renal clearance) that may underlie group differences.^246^

In conclusion, rapid progress in biomarker development is providing opportunities to improve AD diagnosis, but using these advances to benefit patients requires an understanding of the limitations of these tests. Further, it is essential to consider if, when, and how to communicate these potentially life-changing results.

## Clinical implementation

This narrative review has shown that many variables need consideration when assessing associations between plasma biomarkers and NPS. Heterogeneity in NPS definitions and case ascertainment, heterogeneity in cognitive status or syndrome, and heterogeneity in biomarker assays and platforms all contribute to heterogeneity in findings. Further, different approaches are required depending on the purpose of the biomarker—diagnostic biomarkers may differ from prognostic or monitoring biomarkers. Clarity of purpose will be essential for clinical implementation.

The AD Core 1 biomarkers (Aβ, p-tau) are likely the first to be used in the assessment of NPS in clinic, following their use for assessment of cognitive impairment. However, clinicians will first need to be comfortable using the biomarkers for NPS, which will likely occur after experience and comfort with the use of these biomarkers for cognition.

This manuscript serves as a reference and starting point by identifying strengths and opportunities in the field. Some clinical implications may be derived from the available evidence based on contexts of use:^247^

- Plasma biomarkers will hardly replace standardized neuropsychiatric assessments for diagnosis of NPS. Nonetheless, they could be useful to select specific cohorts for clinical trials based on the expected outcomes.
- Plasma biomarkers could be potentially useful to forecast behavioral decline, and for prognostic predictions particularly concerning responses to specific therapeutic interventions. Once validated for target engagement, they can also be used to monitor therapeutic response and assess efficacy.
- Finally, plasma biomarkers could be used to detect adverse reactions or safety concerns of a behavioral therapy. In the general population, they might potentially be used to detect the behavioral effects of exposure to environmental agents. All these implications will depend upon well-grounded research aiming to investigate associations of plasma biomarkers with meaningful behavioral outcomes.

## Limitations and directions for future research

This review aimed to summarize evidence on a topic that has not yet been widely or systematically studied. As this area of study is quite nascent, and the literature scant, we conducted a narrative review rather than a systematic review. We leveraged the subject matter expertise of the author group and collective knowledge of the field to guide us. The authors implemented NPS and dementia-relevant search terms, supplemented by a review of reference lists, and their own knowledge of the field. We tried to include all relevant articles that were available. Nevertheless, the lack of a systematic review methodology should be considered a limitation of this work. In addition, the assessed studies had different inclusion and exclusion criteria, used different protocols to assess NPS and plasma biomarkers, combined patients at different dementia stages, and had diverse sample sizes (often small), thus making it challenging to reach more comprehensive conclusions.

Most of the included studies were cross-sectional, a result of the fact that this topic has not received the required attention from large, multi-center studies. In addition, the scientific community has provided much more attention to AD in comparison to other dementia syndromes, resulting in a discrepant proportion of articles related to AD specifically. Future research should address the relevance of plasma biomarkers in longitudinal studies of NPS, and consider the etiological diversity of dementia.

Standardization of pre-analytical protocols must be widely adopted to harmonize methods and ensure the reliability of research results.^248^ Variability in the associations of plasma biomarkers with neuropathology is still widespread due to the non-adoption of proper control of pre-analytical factors that may interfere with biomarker measurements. Additionally, the summarized studies of non-core AD biomarkers should be viewed as suggestive at this point due to use of non-standardized assays. Additional research, especially longitudinal studies are required.

Plasma p-tau species are non-invasive and reliable indicators of both amyloid and tau pathology and more accurate than plasma amyloid biomarkers to predict conversion to dementia.^249^ However, their negative predictive value is still not high enough. Different plasma analytes are differentially informative of brain events at various stages of AD. Much still needs to be learned about the processes that control both the production and clearance of analytes when dealing with a biofluid biomarker that affects the interpretation of steady-state concentrations. The field requires a broader variety of plasma biomarkers that are both sensitive and specific to neuropathology.^20,250^ Relatedly, the science continues to evolve. For example, CSF Aβ relationships with other biomarkers of AD-related processes, such as those for oxidative damage, are complex, nonlinear, and not fully understood.^251–254^ Similarly, in *PSEN1* variant carriers and in patients with DLB, a pleiotropic change is suggested at the time of cognitive failure.^255,256^ Future studies need to incorporate behavior into this type of modeling.

Finally, implementing widely available diagnostic criteria for NPS and differentiation from pre-existing psychiatric pathology is essential for proper associations with plasma biomarkers. Standardizing neuropsychiatric assessments for detecting NPS consistently may improve their detection and differential diagnoses with primary psychiatric syndromes, align clinical and research procedures, reduce costs, and benefit patients, caregivers, and healthcare systems with the cost-effective standard of care and research for NPS.

## Next steps

With the ongoing efforts by the scientific community to understand AD (and other NDDs) as a biological construct^2^ to facilitate therapeutic development, clinicians need to translate that information into easily understandable recommendations for their patients. In clinical studies, researchers should communicate closely and follow up with their participants.^257^ Autonomous decision-making in people with or at risk for dementia depends on promoting understanding by clearly delineating evidence of benefits, risks, and gaps in knowledge,^258^ including the translational aspects of plasma biomarkers of NPS.

Consistent selection of neuropsychiatric tests and their harmonization,^259^ as has been done with neuropsychological tests,^260^ might enable the use of mechanistically relevant biomarkers and treatment in line with their demonstrated informative or therapeutic value, with clear uniformity across different centers. More original studies with validated and harmonized methods to assess both NPS and plasma biomarkers in diverse cohorts are required,^2^ including less-studied biomarkers of neuroinflammation and endothelial dysfunction.^261^ Other factors that may also affect NPS measurement, such as informant characteristics for informant-rated tools, should be considered.^262^ Agitation, apathy, depression, and psychosis are the most frequently studied NPS, for which there are syndromic criteria, though many others could be included in study protocols with large samples. Such studies should address all NPS in different stages of different dementia syndromes and not only more frequent symptoms.

Important questions remain open, highlighting how much needs to be done with regards to biomarker development in this space. Whether some pre-existing or co-morbid condition underlying NPS symptoms significantly contributes to the trajectory of AD, or whether AD pathology occurring years before cognitive symptoms emerge affects processes that determine NPS are important questions to pursue further.

The associations of some less studied plasma biomarkers with cognition and neuropathology have already been demonstrated in the literature, but no studies with NPS may be currently found. These include circulating microRNAs^263^ and extracellular vesicles^264^ which should also be the target of future studies with behavioral associations.

Longitudinal studies that find associations between NPS and plasma biomarkers throughout the AD continuum while considering the presence of MBI are in high demand. These studies could suggest mechanistic relationships to elucidate how NPS and neuropathology evolve over time, and address if proper therapy may modify such relationships. Systematic reviews that identify, select, synthesize, and appraise all high-quality research evidence relevant to the associations of NPS with plasma biomarkers will be needed when more original studies are available. Actionable items have been drafted to achieve these goals (Table 2).

**Table 2.** Actionable items.

Research Goals	Harmonization of protocols for neuropsychiatric assessment and biomarker studies (and particularly considering pre-analytical factors that may interfere with such assessments) is essential to reach more comprehensive conclusions.
	Biomarker studies that assess associations with neuropsychiatric syndromes must consider potential sex differences and be conducted in cohorts with racial and ethnic diversity, as well as diverse educational and income levels.
	Longitudinal studies with large cohorts that aim to investigate associations between neuropsychiatric syndromes and plasma biomarkers throughout the Alzheimer's disease continuum while considering the presence of mild behavioral impairment may help elucidate cause-and-effect relationships.
	Systematic reviews assessing associations of plasma biomarkers with neuropsychiatric syndromes will be needed when more studies on the field are available.
	The associations of some less studied plasma biomarkers with behavior should also be the target of future studies.
Clinical Goals	Standardization of neuropsychiatric assessments may improve the detection of neuropsychiatric syndromes and differential diagnoses with primary psychiatric syndromes, thus reducing costs and benefitting patients, caregivers, and healthcare systems.
	A broader variety of plasma biomarkers that are both sensitive and specific to neuropathology is required.
	Communication and support must be provided to patients when sharing biomarker information due to potential distress when assessing neuropsychiatric prognoses (pre-test counseling and post-test explanations).
	Objective associations of plasma biomarkers with neuropsychiatric syndromes can be important not only for patient information but also for use in clinical trials and for prediction of behavioral status at different timepoints in the continuum of dementia syndromes.

## Implications and conclusion

The pathogenesis of behavioral symptoms is guided by neurochemical consequences of environmental factors and genetically-mediated proteinopathies. Objective associations of plasma biomarkers with NPS can be important not only for patient information during routine follow-up but also for use in clinical trials and for prediction of behavioral status at different timepoints in the continuum of dementia syndromes.

The etiology of NPS and their underlying conditions should be determined with appropriate clinical workup, including plasma biomarkers that are minimally invasive by nature. This is important, as treatment is likely to vary depending on the underlying etiology. Nevertheless, it remains to be determined if treatment of later-life emergent and persistent NPS in preclinical and prodromal disease will change the disease course and delay or prevent incident behavioral and cognitive decline and dementia.
